# Rare Case of Focal Organising Pneumonia With Extension to Interlobar Space and Adjacent Lobe Initially Suspected as Lung Cancer

**DOI:** 10.1002/rcr2.70144

**Published:** 2025-04-13

**Authors:** Sachie Koike, Takayuki Shiina, Keiichiro Takasuna, Akane Kato, Koudai Komatsu, Toshitsugu Nakamura

**Affiliations:** ^1^ Department of Thoracic Surgery Ina Central Hospital Ina Japan; ^2^ Division of General Thoracic Surgery, Department of Surgery Shinshu University School of Medicine Matsumoto Japan; ^3^ Department of Respirology Ina Central Hospital Ina Japan; ^4^ Department of Pathology Ina Central Hospital Ina Japan

**Keywords:** adjacent lobe, focal organising pneumonia, *Fusobacterium nucleatum*, interlobar space, lung cancer

## Abstract

Organising pneumonia is histopathologically characterised by inflammatory granulation tissue plugs within the lumen of the small airways, extending into the alveolar ducts and airways. Focal organising pneumonia is an organising pneumonia that presents as a solitary nodule or mass on computed tomography. This type of organising pneumonia is sometimes initially suspected to be lung cancer. We report a rare case of focal organising pneumonia that extended to the interlobar space and adjacent lobe and was initially suspected of lung cancer with interlobar pleural invasion. This case of focal organising pneumonia may have been induced by *Fusobacterium nucleatum* infection. This is the first report of a focal organising pneumonia extending to the interlobar space and adjacent lobe.

## Graphical abstract

We report a rare case of focal organising pneumonia (FOP) that was initially suspected to be lung cancer with interlobar pleural invasion and extension to the interlobar space and the adjacent lobe.

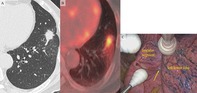

## 1 Introduction

Organising pneumonia (OP) is a pulmonary clinicopathological syndrome, and some of the lesions are the result of a reaction to noxious stimuli such as infections, drugs, and radiation therapy; or endogenous factors such as collagen vascular diseases [1]. OP is histopathologically characterised by inflammatory granulation tissue plugs within the lumens of small airways extending into the alveolar ducts and airways [1, 2]. OP has various radiological features such as ground‐glass attenuation, crazy paving, reverse halo, and multiple nodules [1]. OP occasionally presents as a solitary nodule or mass on computed tomography; this type of OP is called focal OP (FOP). FOP is often initially suspected to be lung cancer [1, 2].

We report a rare case of FOP that was initially suspected to be lung cancer with interlobar pleural invasion and extension to the interlobar space and adjacent lobe.

## 2 Case Report

**FIGURE 1:**
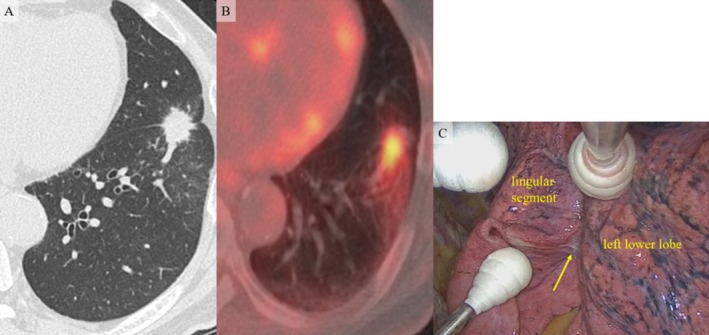
Radiological images and intraoperative findings. (A) Chest computed tomography revealed a 3.0 cm mass with spicula in the left S8 near the interlobar fissure. The mass appeared to invade the lingular segment. (B) The maximal standard uptake value (SUVmax) of ^18^F‐fluorodeoxyglucose positron emission tomography (FDG‐PET) of the mass was 4.49 on the early scan and 5.18 on the delayed scan. (C) Adhesion was revealed between the left lower lobe and the lingular segment. An abscess‐like mass (arrow) was found in the adhered interlobar space.

A 74‐year‐old man with a history of diabetes mellitus and periodontitis was referred to us with a mass in the left lower lobe of the lung on screening chest computed tomography (CT). The results of laboratory tests revealed high haemoglobin A1c (HbA1c) (7.3%) levels; however, other parameters were within normal limits. The white blood cell counts and C‐reactive protein levels were normal, as were the levels of the following tumour markers: carcinoembryonic antigen, squamous cell carcinoma antigen and pro‐gastrin‐releasing peptide. Chest CT revealed a 3.0 cm mass with spicula in the left segment 8 (S8) near the interlobar fissure. The mass appeared to invade the lingular segment (Figure 1A). The maximal standard uptake value (SUVmax) of ^18^F‐fluorodeoxyglucose positron emission tomography (FDG‐PET) of the mass was 4.49 on the early scan and 5.18 on the delayed scan (Figure 1B). We performed bronchoalveolar lavage and transbronchial fine needle aspiration via bronchoscopy (obtained specimen from B8a); however, no malignancy or bacteria or mycobacteria infection was found. We scheduled surgery for lung cancer with interlobar pleural invasion based on these findings.

We found dense adhesion between the left lower lobe and the lingular segment during surgery. An abscess‐like mass was observed in the adhered interlobar space (Figure 1C). We performed wedge resection (resected part of the left lower lobe and the lingular segment) for diagnosis because the mass appeared benign. The postoperative course of the patient was unremarkable.

**FIGURE 2:**
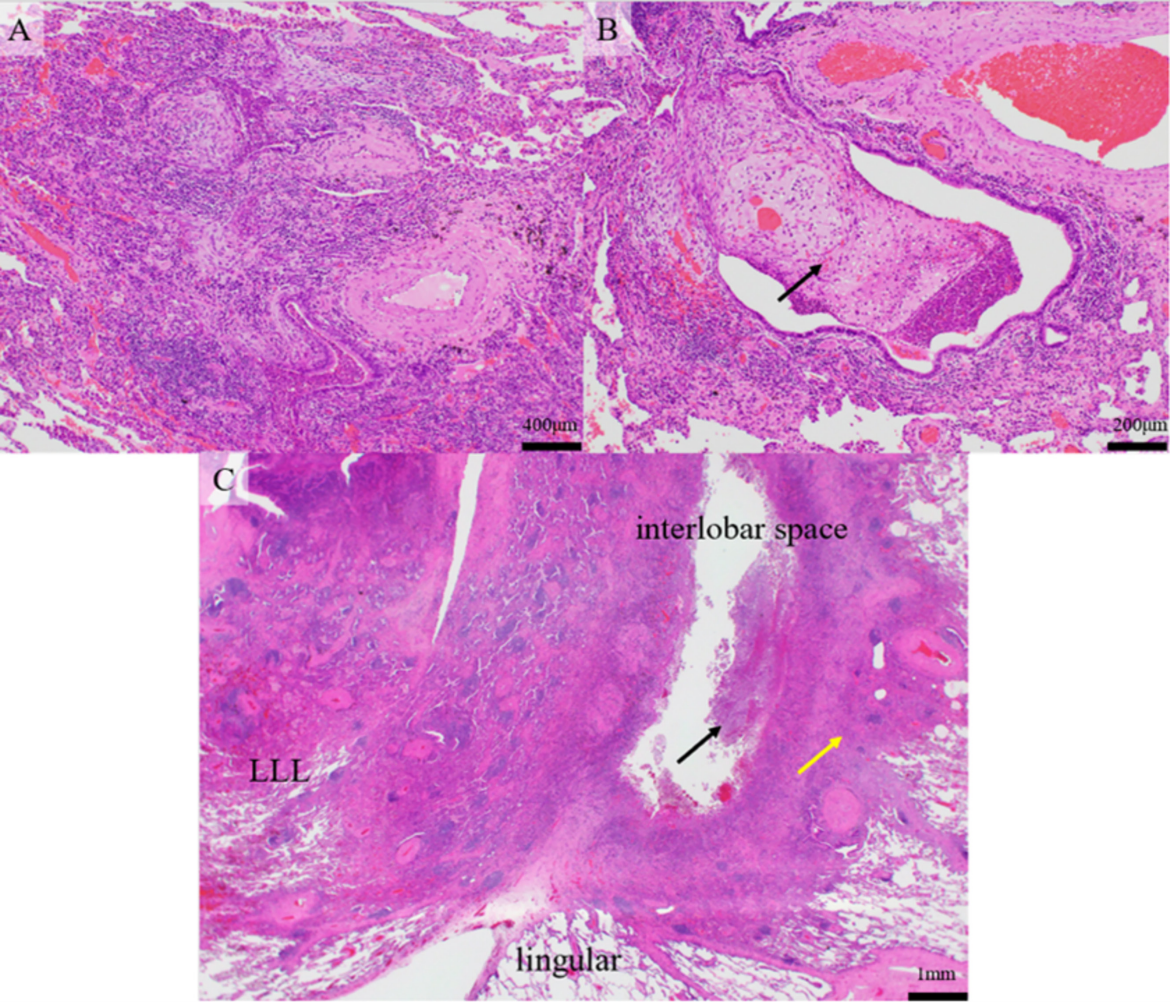
Pathological findings. (A) The alveoli and alveolar ducts were filled with plugs of granulation tissue, which were composed of fibroblasts, and around these areas, lymphocytes and plasma cells infiltrated. The typical characteristics of organising pneumonia were found. (B) A plug of granulation tissue in the bronchiole (Masson body, arrow). (C) Fibrous adhesion was made between the specimen of left lower lobe and lingular segment, and numerous neutrophils infiltrated (black arrow). The inflammatory cells and granulation tissue (organising pneumonia) were extended to lingular segment (yellow arrow). LLL, left lower lobe; lingular, lingular segment.

Pathological examination revealed the inflammatory exudate, which includes numerous neutrophils, filled the lumen of the bronchioles in the left lower lobe specimen. The typical OP characteristics were observed in most areas. The alveoli and alveolar ducts were filled with plugs of granulation tissue composed of fibroblasts, and lymphocytes and plasma cells infiltrated around these areas (Figure 2A,B). Fibrous adhesion was observed between the specimen of the left lower lobe and the lingular segment, with numerous infiltrated neutrophils (Figure 2C). The inflammatory cells and granulation tissue extended into the lingular segment (Figure 2C). *Fusobacterium nucleatum* was detected in a culture of the inflammatory exudate of the specimen. We diagnosed the mass as an FOP that extended to the interlobar space and adjacent lobe, secondary to *Fusobacterium nucleatum* infection, based on these findings. Treatment was terminated given the lack of other sites of bacterial infection.

## 3 Discussion

We initially suspected the FOP as lung cancer because of the extension of the mass to the interlobar space and adjacent lobe, the relatively high and increased SUVmax according to FDG‐PET, and the bronchoscopy results.

FOP extending to the interlobar space or adjacent lobe is extremely rare. This is the first report of FOP extending outside the lung visceral pleura. We initially diagnosed the mass as lung cancer based on invasive radiological findings, and the patient underwent surgical treatment.

FDG‐PET is an imaging modality that differentiates benign from malignant focal pulmonary abnormalities, with SUVmax > 2.5 suggesting malignancy [3]. However, benign lesions can be noted with SUVmax > 2.5 [3, 4]. Tateishi et al. reported that the average SUVmax for OP was 2.95 ± 0.13 [4]. Dual time point FDG‐PET is used to increase diagnostic accuracy, involving a scan at two sequential time points and has very high sensitivity and specificity. An increase in the SUVmax between the early and delayed scans suggests a malignant lung tumour [3]. In the present case, the SUVmax of the mass was 4.49 and 5.18 on the early and delayed scans, respectively. These values were higher than the average for OP, and an increase in the SUVmax was observed between scans. These images hindered the differentiation of the lesion from lung cancer.

In the present case, *F. nucleatum* was detected in the culture of the inflammatory exudate from the resected specimen. This suggested that the FOP was induced by *F. nucleatum* infection. *F. nucleatum* is an anaerobic oral commensal organism often associated with inflammatory bowel disease, adverse pregnancy outcomes, respiratory tract infections, and Lemierre's syndrome [5]. Anaerobes can be difficult to isolate and culture; the incidence of anaerobes is only 20% with conventional cultivation techniques [5]. In the present case, surgical treatment would not have been performed if a bacterial infection were detected upon bronchoscopy. The anaerobic characteristics of the pathogen may have affected the diagnosis.

In conclusion, we present a rare case of FOP that was initially suspected to be lung cancer with interlobar pleural invasion and extension to the interlobar space and adjacent lobe. The interlobar extension, FDG‐PET images, and results of preoperative bacterial cultivation were the causes of this misdiagnosis.

## Ethics Statement

This study was approved by the ethics review board of Ina Central Hospital, Ina, Nagano, Japan (approval number: 24‐17). We obtained comprehensive written informed consent from the patient.

## Conflicts of Interest

The authors declare no conflicts of interest.

## Data Availability

The data underlying this article cannot be shared publicly for protecting privacy of individuals that participated in this study. The data may be shared on reasonable request to the corresponding author after an additional approval by the Institutional Review Board of Ina Central hospital, Japan.
